# One year clinical outcomes of Rutherford 6 chronic limb threatening ischemia patients undergoing lower limb endovascular revascularisation from Singapore

**DOI:** 10.1186/s42155-022-00306-1

**Published:** 2022-07-06

**Authors:** Rui En Lee, Ankur Patel, Shereen Xue Yun Soon, Sze Ling Chan, Charyl Jia Qi Yap, Sivanathan Chandramohan, Luke Hsien Ts’ung Tay, Tze Tec Chong, Tjun Yip Tang

**Affiliations:** 1 Yong Loo Lin School of Medicine, National University of Singapore, 10 Medical Dr, Singapore, 117597 Singapore; 2 Department of Vascular Interventional Radiology, Singapore General Hospital, Singapore, Singapore; 3 Duke-NUS Graduate Medical School, 8 College Rd, Singapore, 169857 Singapore; 4 Health Services Research Centre, SingHealth, Academia, Ngee Ann Kongsi Discovery Tower Level 6, 20 College Road, Singapore, 169856 Singapore; 5 Department of Vascular Surgery, Singapore General Hospital, Level 5; Academia, 20 College Road, Singapore, 169856 Singapore

**Keywords:** Rutherford 6, CLTI, Peripheral arterial disease, Endovascular, Revascularisation, VQI

## Abstract

**Background:**

Percutaneous transluminal angioplasty (PTA) is widely used as a first-line revascularisation option in patients with chronic limb threatening ischemia (CLTI). This study aimed to evaluate the short-term endovascular revascularisation treatment outcomes of a cohort of Rutherford 6 (R6) CLTI patients, from a multi-ethnic Asian population in Singapore.

Patients with R6 CLTI who underwent endovascular revascularisation from June 2019 to February 2020 at Singapore General Hospital, a tertiary vascular centre in Singapore, were included and followed up for one year. Primary outcome measures included number and type of reinterventions required, 3-, 6- and 12-month mortality, 6- and 12-month amputation free survival (AFS), wound healing success and changes in Rutherford staging after 3, 6 and 12 months.

**Results:**

Two hundred fifty-five procedures were performed on 86 patients, of whom 78 (90.7%) were diabetics, 54 (62.8%) had coronary artery disease (CAD) and 54 (62.8%) had chronic kidney disease (CKD). 42 patients (48.8%) required reintervention within 6 months. Multivariate analysis revealed that the presence of CAD was a significant independent predictor for reintervention. Mortality was 15.1%, 20.9% and 33.7% at 3, 6 and 12 months respectively. AFS was 64.0% and 49.4% at 6 and 12 months. Inability to ambulate, congestive heart failure (CHF), dysrhythmia and CKD were significant independent predictors of lower 12-month AFS.

**Conclusions:**

PTA for R6 CLTI patients was associated with relatively high mortality and reintervention rates at one year. CAD was an independent predictor of reintervention. More research is required to help risk stratify which CLTI patients would benefit from an endovascular-first approach versus conservative treatment or an immediate major lower extremity amputation policy.

## Background

Chronic limb threatening ischaemia (CLTI) is a clinical syndrome that represents the most advanced stage of peripheral artery disease (PAD) and is defined as rest pain or lower limb ulceration for more than two weeks duration (Conte et al. 2019). These patients are at high risk of major lower extremity amputation (LEA) and premature death (Duff et al. 2019). The one-year risk of major LEA in patients with CLTI exceeds 15–20% and the five-year all-cause mortality rate is approximately 50% (Duff et al. 2019).

The Rutherford staging system for CLTI describes six clinical categories of lower extremity ischemia, from Rutherford 0 (R0) patients who are asymptomatic to R6 patients who have major tissue loss extending above the trans-metatarsal level, with a functional foot that is regarded as non-salvageable (Rutherford et al. 1997). R6 patients are regarded in literature as severe CLTI patients with little to no revascularisation or pharmacological options as they have presented too late or are too advanced in their disease (Sprengers et al. 2010). Ultimately, major amputation may be the only option. Amongst patients with CLTI, R6 patients have the highest major LEA and mortality rates despite undergoing the same treatment as R4 and R5 patients (Brodmann et al. 2020). As R6 patients carry the worst clinical prognosis (Sprengers et al. 2010), it is important that these patients are prioritised in studies addressing lower limb revascularisation options and outcomes.

R6 CLTI patients tend to also carry multiple comorbidity burdens. Advanced cerebrovascular disease (CVD) and coronary artery disease (CAD) are more frequent in CLTI patients as compared to patients with less severe PAD. Chronic kidney disease (CKD) and end stage renal failure (ESRF) are also associated with an increased risk of CLTI and cardiovascular mortality (Angelantonio et al. 2010). Patients with known CKD have a 1.8 times higher frequency of severe PAD (R5 or R6) as compared to those without (Lüders et al. 2016). Additionally, diabetes mellitus (DM) increases the risk of developing CLTI four-fold (Becker et al. 2011). Among patients who had undergone endovascular therapy for CLTI, the presence of DM further increased the risk of major LEA and myocardial infarction (Lilja et al. 2020), signifying that CLTI in diabetics is more challenging to treat. In Asia, the burden of PAD and DM is already projected to increase, and the majority of Asian patients with PAD may have concomitant DM (Kawarada et al. 2018). Similarly, the number of R6 patients in Asia and in Singapore is also set to increase.

These demographic trends in co-morbidities serve to illustrate the vulnerability of R6 CLTI patients, and the increased likelihood of poorer outcomes as a result of these co-morbidities. Despite the clinical significance of this group of patients, guidelines on the preferred treatment options and detailed studies of treatment outcomes based on current protocols are lacking in current literature. For patients with advanced CLTI disease patterns, such as those of R6 patients, angiography may not yield a patent artery distal to the affected part (Conte et al. 2019). In patients with advanced tissue loss, endovascular intervention via percutaneous transluminal angioplasty (PTA) is associated with poorer limb salvage and a significant proportion of them require early major amputation (Lee et al. 2020). Similarly, patients with complicated anatomic patterns of occlusion and increased limb threat may not benefit from a universal endovascular-first approach. Primary bypass surgery had better outcomes than secondary bypass surgery following failed endovascular interventions (Conte et al. 2019; Bradbury et al. 2010; Iida et al. 2017). Thus, the revascularisation technique has to be carefully selected before proceeding to surgery.

This study aims to define the one-year endovascular revascularisation treatment outcomes of a cohort of R6 patients from a multi-ethnic Southeast Asian population in Singapore.

## Methods

### Patients

This study retrospectively reviewed patients with R6 CLTI who underwent endovascular revascularisation procedures at Singapore General Hospital, a tertiary vascular centre in Singapore. Patients from June 2019 to February 2020 were included, and the patients were followed-up for a period of 12 months. Written consent was obtained for their participation in this study. The patients’ data were collected via the Vascular Quality Initiative (VQI) (Woo et al. 2013) database, and they were each identified using a unique VQI identifier. In addition, their surgical case notes and health records were reviewed on the hospital’s electronic medical record system. The SingHealth Centralised Institutional Review Board approved this study (CIRB number: 2018/2995).

Pre-intervention variables collected included patient demographics, co-morbidities, medications and results from pre-operative investigations. For each patient, wound factors evaluated included the site and length of the lesion, Rutherford classification score (Rutherford et al. 1997), Wound Ischemia and foot Infection (WIfI) score (Mills et al. 2014), Trans-Atlantic Inter-Society Consensus Document II (TASC II) grade (Norgren et al. 2007), prior interventions and reasons for the current intervention. These wounds were classified according to the lesions that they pertained to. Specific information regarding the extent of the wounds was not obtained.

A lesion was defined as a region of stenosis or occlusion within the target limb secondary to atherosclerotic disease. Lesions were defined as discrete if they were separated by at least 3 cm. Data was collected for one limb per patient, and most had multiple lesions in the same extremity. The lesions that underwent reintervention were counted as separate lesions. Treatment factors recorded included the type of treatment used and technical success of revascularisation. Technical success post-intervention was defined as less than or equal to 30% residual stenosis within the target vessel. Post-intervention outcomes measured comprised the number and type of reinterventions required, 3-, 6-, and 12-month mortality, 6- and 12-month amputation free survival, wound healing success as well as changes in Rutherford staging after 3, 6 and 12 months.

### Procedure

The risk profiles of the patients were optimised before each intervention. Each lower limb angioplasty procedure was performed by an attending vascular surgeon or vascular interventionalist under local, regional or general anaesthesia depending on patient and procedural factors. Standard digital subtraction angiography techniques were utilised with minimal use of contrast agents. Prior to intervention, intra-arterial heparin (1000–5000 IU) was routinely administered. The choice of treatment type – plain or drug coated balloons, stents or atherectomy – was decided by the vascular specialist. The technique applied involved crossing the lesion successfully with a wire, dilating it with a balloon with the same diameter as the normal vessel and leaving the balloon in-situ for at least 2 min. All patients received at least single-agent antiplatelet therapy (aspirin and/or clopidogrel) and statin therapy post revascularisation for at least six months. Platelet aggregation studies were not done to determine if a patient would respond to a single antiplatelet agent. Adherence is rather debatable in our population, as evidenced by a recent paper from our institution (Tang et al. 2021a). Other medical management options such as low dose rivaroxaban (Xarelto) as per the Voyager PAD study (Bonaca et al. 2020) recommendations were left at the discretion of the treating specialist, but these only involved a minority of the patients. Activated clotting times were not routinely checked after heparinisation during lower limb angioplasty.

Detailed procedural information has previously been published (Tay et al. 2020). All patients were followed-up in the vascular outpatient clinic at 3, 6 and 12 months after the initial intervention to assess wound healing progression and Rutherford staging. Preoperative duplex imaging for angiographic planning was performed on all patients requiring reintervention.

Singapore General Hospital has a primary endovascular-first policy in view of the patients’ co-morbidities and frailty. Open bypass surgeries are usually reserved for younger, more surgically fit patients with long femoral-popliteal occlusions with good inflow and outflow vessels and a good venous conduit, or for patients who have failed endovascular intervention. In our local context, many patients prefer minimally invasive surgical methods as compared to open surgery.

### Cohort and variable definitions

The Rutherford Classification was used to define the extent of CLTI (Rutherford et al. 1997). The WIfI score consists of three components, Wound (W), Ischemia (I) and Foot Infection (fI) (Mills et al. 2014). The mean WIfI score was calculated using the average of the numerical sum of the three components (W + I + fI). Major LEA was defined as amputation above the level of the ankle, while minor LEA was defined as amputation restricted to the toes or at the level of the forefoot.

### Statistical analysis

Descriptive analyses of demographic and clinical variables were performed based on patients and procedures as the unit of analysis, as appropriate. Survival probability was calculated based on the individual, from the date of the first operation. Univariate analysis was conducted on categorical outcomes using logistic regression. Variables with *p* < 0.05 were selected for multivariate analysis. The association between demographic and clinical variables with survival on the patient level was also analysed using a Cox proportional hazards model. Similarly, univariate analysis was performed, and variables with *p* < 0.05 were entered into a stepwise multivariable Cox regression. All analyses were conducted using R version 3.5.1 (R: A 2020).

## Results

Eighty six patients (255 lesions) were included. 54/86 (62.8%) were male and mean age was 66.0 ± 10.6 years old. 78/86 (90.7%) had hypertension, 78/86 (90.7%) DM, 54/86 (62.8%) CAD, 20/86 (23.3%) CVD and 54/86 (62.8%) CKD. Baseline median toe pressure was 40 (interquartile range (IQR) 24–51) mmHg. 63/86 (73.2%) patients were ambulant either independently or with assistance pre-morbidly. Demographic data and medical co-morbidities are summarised in Table 1.

**Table 1:** Patient Demographics and Prior Interventions

***Patient Demographics***	Number of patients (*n* = 86)	Percentage (%)
Mean Age, years (SD)	66.0 ± 10.6	
Mean BMI, kg/m^2^ (SD)	24.6 ± 4.7	
Gender		
Male	54	62.8
Female	32	37.2
Ethnic Group		
Chinese	60	69.8
Malay	12	14.0
Indian	13	15.1
Eurasian	1	1.2
Smoking Status		
Smoker	15	17.4
Non-smoker	54	62.8
Ex-smoker	16	18.6
Co-morbidities		
Diabetes	78	90.7
Hypertension	78	90.7
CAD^a^	54	62.8
CVD^b^	20	23.3
Dysrhythmia	22	25.6
COPD^c^	4	4.7
Congestive Heart Failure	16	18.6
Kidney impairment	54	62.8
ESRF^d^ on dialysis	38	44.2
CKD^e^ (not on dialysis)	16	18.6
or on transplant		
Pre-operative Mobility Status		
Independently ambulant	31	36.0
Ambulatory with assistance	32	37.2
Pre-operative Investigations	40 (IQR 24–51)	
Median toe pressure (mmHg)		
***Prior Interventions***		
Any intervention	72	83.7
Coronary Artery Bypass Graft	14	16.3
Percutaneous Coronary Intervention	15	17.4
Leg arterial bypass/ Endarterectomy/PVI^f^	48	55.8
Femoral Endarterectomy	2	2.3
Amputations		
Minor (toes, forefoot)	65	75.6
Major contralateral (BKA^g^, AKA^h^)	10	11.6

Total: 86 patients; 86 limbs (48 right, 38 left); 255 lesions

^a^CAD: coronary artery disease

^b^CVD: cerebrovascular disease

^c^COPD: chronic obstructive pulmonary disease

^d^ESRF: end stage renal failure

^e^CKD: chronic kidney disease

^f^PVI: peripheral vascular intervention

^g^BKA: below knee amputation

^h^AKA: above knee amputation

72/86 (83.7%) had undergone previous vascular interventions; 15/86 (17.4%) had previously undergone a percutaneous coronary intervention. 65/86 (75.6%) and 10/86 (11.6%) had undergone minor and major contralateral LEA, respectively. The details of prior interventions are summarised in Table 1. Mean baseline WIfI score was 4.3 ± 1.7 (Table 2). Patients who had osteomyelitis or other forms of subcutaneous wound involvement (e.g. abscess, septic arthritis) were given a score of Grade 2 and above for the ‘Infection’ component of their WIfI score. There were 36 such patients. 114/255 (44.7%) lesions were classified as TASC II grade A or B atherosclerotic lesions, and 141/255 (55.3%) were classified as TASC II grade C or D lesions (Table 2). Patients with TASC II grade A or B lesions were still R6 patients, due to a significant neuropathic component in their disease which led to ulceration. The mean lesion length was 12.9 ± 12.0 cm. 85/255 (33.3%) were located in the femoro-popliteal region, 60/255 (23.5%) in the anterior tibial artery, 31/255 (12.2%) in the peroneal artery and 28/255 (11.0%) in the posterior tibial artery. 231/255 (90.6%) lesions achieved technical success. 288/343 (84.0%) and 49/343 (14.3%) procedures conducted were plain balloon and drug coated balloon angioplasties respectively, compared to 4/343 (1.2%) which were stent insertions. Interventional and procedural details can be found in Table 2.

**Table 2:** Interventional and Procedural Details

**Interventional Details**	Number of limbs (*n* = 86)	Percentage (%)
Urgency		
Elective	34	39.5
Urgent	50	58.1
Emergency	2	2.3
Symptoms (Indicated limb)		
Ulcer/Necrosis	64	74.4
Non-healing Amputation	15	17.4
Both Ulcer + Non-healing amputation	6	7.0
Acute Ischemia	1	1.2
WIfI Score (Indicated limb)		
Mean WIfI Score (SD)	4.3 ± 1.7	
Wound		
0 (none)	3	3.5
1 (shallow)	21	24.4
2 (deep)	35	40.7
3 (extensive)	27	31.4
Ischemia (toe pressure, mmHg)		
0 (none)	38	44.2
1 (40–59)	22	25.6
2 (30–39)	10	11.6
3 (< 30)	16	18.6
Infection		
0 (none)	20	23.3
1 (mild)	31	36.0
2 (moderate)	31	36.0
3 (severe)	4	4.7
Clinical Stages (Risk of Amputation)		
1 (very low risk)	9	10.5
2 (low risk)	18	20.9
3 (moderate risk)	18	20.9
4 (high risk)	41	47.7
**Procedural details**	Number of lesions (*n* = 255)	Percentage (%)
**Lesion details**		
Right	142	55.7
Left	113	44.3
Technical success		
Successful (Stenosis ≤ 30%)	231	90.6
Stenosis > 30%	7	2.7
Target Lesion Occlusion	3	1.2
Technical Failure	14	5.5
Mean lesion length (cm)	12.9 ± 12.0	
Location of treated vessel		
Aorto-iliac	11	4.3
Common Femoral Artery (CFA)	3	1.2
Superior Femoral Artery (SFA) + Popliteal	85	33.3
Anterior Tibial Artery (ATA)	60	23.5
Posterior Tibial Artery (PTA)	28	11.0
TP Trunk	16	6.3
Peroneal	31	12.2
Common Plantar Artery	7	2.7
Dorsalis Pedis Artery (DPA)	14	5.5
TASC II Classification		
A	47	18.4
B	67	26.3
C	62	24.3
D	79	31.0
	Number of lesions (*n* = 255)	Percentage (%)
**Treatment type (total number of procedures)**		
Plain balloon	288	84.0
Drug coated balloon	49	14.3
Stent	4	1.2
Atherectomy	2	0.6
Mean number of plain balloons per leg	3.3	
Mean number of drug coated balloons per leg	0.6	

Patients with multiple lesions underwent all initial interventions in the same sitting. Patients whose procedures did not achieve technical success were offered prophylactic forefoot and topical oxygen therapy as a form of conservative treatment. Not to be confused with hyperbaric oxygen therapy, topical oxygen therapy uses a compact battery-powered “oxygen generator” to concentrate atmospheric oxygen and feed pure oxygen through a fine soft tube to a dressing-like “oxygen distribution system”, which is held in place by a conventional dressing (Tang et al. 2021b). Open revascularisation was only performed after endovascular revascularisation had failed. Approximately 10–15% of patients had the option to undergo open revascularisation, as they have multi-level disease with substantial microvascular components affecting runoff. Bypass was offered based on the individual patient’s surgical risk and outlook, but the majority of our patients were diabetic, did not have good vein conduits and had poor co-morbids. Hence, they were poor candidates for open revascularisation surgery. Locally, families and patients are usually not keen for open surgery and the majority will try conservative therapy using local wound adjuncts, topical oxygen and or hyperbaric oxygen therapy.

3 patients were lost to follow-up between 6 and 12 months. Hence, the total number of patients was 83 at 12 months. 42/86 (48.8%) and 46/83 (55.4%) patients required reintervention within 6 and 12 months following the initial revascularisation respectively (Table 3), either a revascularisation or amputation (minor or major) procedure. If patients required a reintervention, it was either for the same lesion that was previously treated, or for new de novo lesions. 23/86 (26.7%) and 27/83 (32.5%) patients underwent a revascularisation procedure within 6 and 12 months. Overall, the target lesion revascularisation rate was 62/255 (24.3%). Notably, most (70/77) of the reinterventions took place within the first 6 months. Details of the post-intervention outcomes at 6 and 12 months can be found in Table 3.

**Table 3:** Post-intervention Outcomes at 6 and 12 Months

**At 6 months**	Number of patients (*n* = 86)	Percentage (%)
Number of patients requiring (in 6 months)		
Reintervention (any)	42	48.8
Revascularisation	23	26.7
Minor amputation	18	20.9
Major amputation	16	18.6
BKA	13	15.1
AKA	3	3.5
Death		
After 3 months	13	15.1
After 6 months	18	20.9
Amputation-free Survival		
After 6 months	55	64.0
Wound Healing		
Healed after 3 months	8	9.3
Healed after 6 months	23	26.7
Rutherford Staging after 3 months		
Stage 0	29	33.7
Stage 5	27	31.4
Stage 6	30	34.9
Rutherford Staging after 6 months		
Stage 0	49	57.0
Stage 5	22	25.6
Stage 6	15	17.4
Reinterventions (any) in 6 months		
Total number	70	
Revascularisation procedures	36	
Minor amputations	21	
Major amputations	16	
**At 12 months**	Number of patients (*n* = 83)^a^	Percentage (%)
Number of patients requiring (in 12 months)		
Reintervention (any)	46	55.4
Revascularisation	27	32.5
Minor amputation	20	24.1
Major amputation	19	22.9
BKA	16	19.3
AKA	3	3.6
Death		
After 12 months	28	33.7
Amputation-free Survival		
After 12 months	41	49.4
Wound Healing		
Healed after 12 months	34	41.0
Rutherford Staging after 12 months		
Stage 0	74	89.2
Stage 5	6	7.2
Stage 6	6	7.2
Reinterventions (any) in 12 months		
Total number	77	
Revascularisation procedures	41	
Minor amputations	23	
Major amputations	19	
Target lesion revascularisation rate (out of 255 lesions)	62	24.3
Median time to (days)		
Reintervention (any)	34	
Revascularisation	80	
Minor amputation	10.5	
Major amputation	81	

^a^Between 6 and 12 months, 3 patients were lost to follow-up. The total number of patients was taken to be 83 at 12 months

The presence of CAD (*P* = *0.02*) predicted a decreased time to any reintervention. Multivariate analysis showed that CAD (*P* = *0.0015*) was a significant independent predictor for reintervention. Specifically, the presence of CVD (*P* = *0.0031*) was found to be a significant independent predictor for minor LEA, while the inability to ambulate pre-operatively (*P* = *0.021*) was also found to be a significant independent predictor for major LEA. 19/86 (22.1%) patients underwent major LEA in 12 months, and the median time to event was 81 (IQR 18.5–127.5) days.

**Fig. 1:**
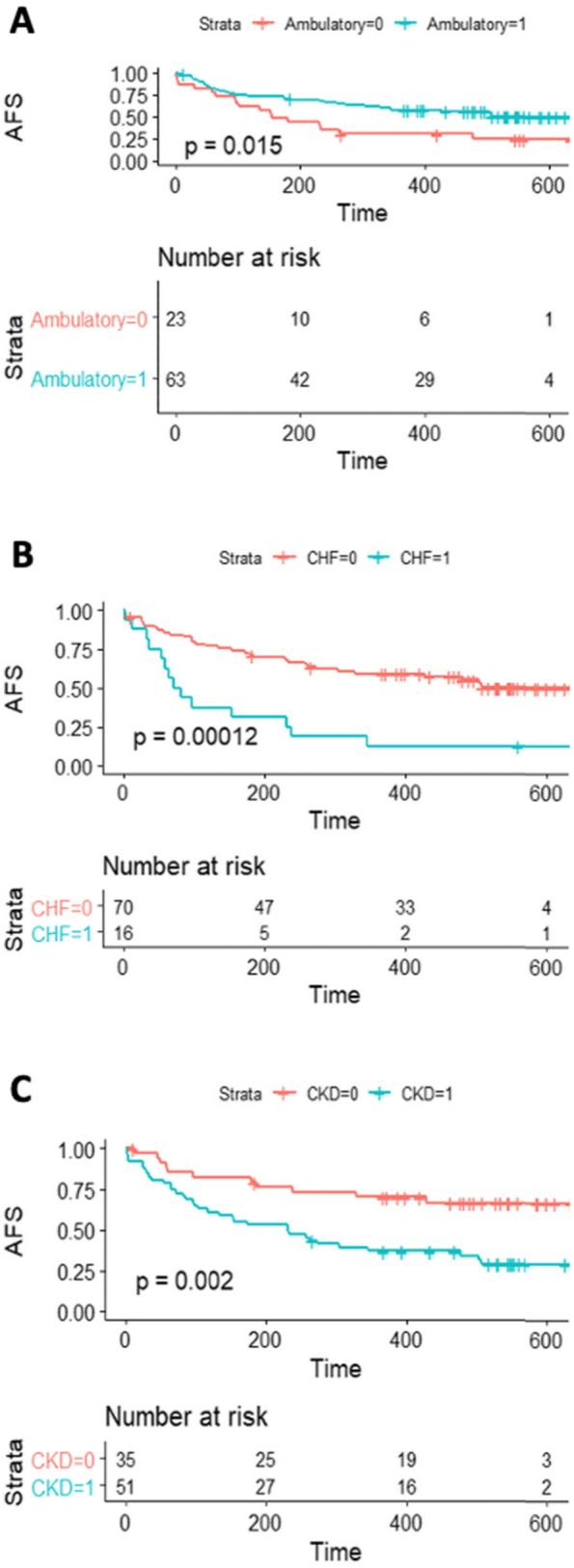
Kaplan–Meier survival curves of (**A**) ambulatory status, (**B**) congestive heart failure and (**C**) chronic kidney disease, independent predictors of lower 12-month AFS

Mortality was (13/86) 15.1%, (18/86) 20.9% and (28/83) 33.7% at 3, 6 and 12 months respectively. The median survival time was 106 (IQR 56–242.8) days. Deaths happened largely from infection such as pneumonia and sepsis, or major adverse cardiovascular events (MACE); underlying co-morbidities significantly contributed to the deaths. AFS was (55/86) 64.0% and (41/83) 49.4% after 6 and 12 months respectively. Time-to-event analysis showed that inability to ambulate preoperatively (*P* = *0.015*), congestive heart failure (CHF) (*P* < *0.001*), and CKD (*P* = *0.002*) were independent predictors of lower 12-month AFS (Fig. 1a, b, c).

Excluding the patients who were no longer alive at the time, 8/86 (9.3%) patients had fully healed wounds at 3 months, while 23/68 (33.8%) and 34/58 (58.6%) patients experienced complete wound healing at 6 and 12 months respectively. For patients who required surgical debridement or digital amputation, wound healing was only documented after these procedures were completed and the patients had recovered from them. Univariate analysis of 3- and 6-month wound healing outcomes showed that CKD predicted a poorer 3-month wound healing outcome (*P* = *0.03*), while CVD predicted a poorer 6-month wound healing outcome (*P* = *0.03*).

30/86 (34.9%), 15/86 (17.4%) and 6/83 (7.2%) patients had R6 wounds at 3, 6 and 12 months respectively. CKD predicted poorer 3- and 6-month Rutherford scores (*P* = *0.01 and P* = *0.02 respectively*).

## Discussion

Defined by major tissue loss and gangrene, R6 is the most severe form of CLTI (Rutherford et al. 1997). Arterial occlusive disease in R6 patients is often so severe that their lower extremities are termed “desert feet” (Kim et al. 2021), which puts these patients at high risk for major amputation due to the lack of effective conventional revascularisation options and presence of microvascular disease. Mortality in R6 patients is also high (33.7% in 12 months). In a Japanese population, 47% had cardiovascular causes of death, including heart failure, acute myocardial infarction and ventricular fibrillation (Soga et al. 2014). R6 disease has been found to be a more significant predictor of 2-year mortality (OR = 3.4) as compared to R5 disease (OR = 1.9) (Soga et al. 2014).

R6 disease has been found to be an independent predictor of delayed wound healing after endovascular therapy (Shiraki et al. 2015), with or without the presence of a heel ulcer (Azuma et al. 2012). Just as the complete epithelialisation of all wounds is a measure of the successful treatment of limbs with ischemic wounds (Azuma et al. 2012), non-healing wounds signify poorer outcomes. After 6 months, 82.6% of wounds in our study were no longer R6 wounds, but only 26.7% experienced complete wound healing. CLTI patients with DM or ESRF have twice the risk of wound healing failure as compared to CLTI patients without (Kawarada et al. 2018; Azuma et al. 2012). CKD affects wound healing by delaying the rate of granulation and reducing cell proliferation rates (Maroz and Simman 2013). The median ulcer healing time of ESRF patients with R6 disease was approximately three times that of ESRF patients with R5 disease (Azuma et al. 2012). In our cohort, high rates of DM and ESRF patients on dialysis, together with R6 disease, compounded the negative effects on wound healing rates.

Moreover, the clinical profile of R6 CLTI patients plays a large role in affecting revascularisation outcomes. Since CLTI is often caused by multi-level occlusive atherosclerotic disease, CLTI patients share the same risk factors as patients with atherosclerosis (Becker et al. 2011), such as DM and CAD (Hurst et al. 2021). There is a higher incidence of advanced CVD and CAD in CLTI patients as compared to patients with less severe PAD (Becker et al. 2011). It has been found that the incidence of co-morbidities, proportion of severely diseased lesions and proportion of patients with tissue loss were higher in a Singaporean CLTI population than that of endovascular Western cohorts from the SVS VQI registry (Soon et al. 2021). However, 6-month AFS in R6 patients following endovascular treatment in Western populations has been reported to be approximately 62–66%(Rocha-Singh et al. 2012; Mustapha et al. 2019), which is comparable to that of our study cohort (64.0%). 6-month mortality rate was 27% (Mustapha et al. 2019), versus 20.9% in our cohort. These results suggest that adopting an endovascular-first, rather than an open bypass strategy seems to yield comparable results in Singaporean R6 patients compared to those in Western populations.

According to the recent CIRSE standard of practice (2021) on below-the-knee (BTK) revascularisation, though POBA remains the first line treatment for long lesions, drug-eluting stents are regarded as "superior in terms of patency, target lesion revascularisation, Rutherford improvement and wound healing at 1-year follow-up, compared to bare metal stenting or plain balloon angioplasty" (Spiliopoulos et al. 2021). While it may appear that our treatment decisions are rather conservative in nature (1.2% stent vs 14.3 drug-coated balloon), it is important to note that Asian blood vessels in general are also smaller than our Caucasian counterparts, thus the treatment of the imminent and subsequent occlusion or in-stent restenosis will be more challenging than a re-occlusion of a native vessel. Further, majority (33.3%) of our lesions were location in the SFA-popliteal region where stenting would present with higher risks of stent fracture and loss of patency.

Revascularisation relieves ischemia from flow-limiting lesions caused by CAD (Libby and Theroux 2005). In balloon angioplasty, shear stress during balloon inflation triggers vascular inflammation, and arterial remodelling and neointimal hyperplasia post-angioplasty cause restenosis (Nakatani et al. 2003). Revascularisation of CAD causes secondary changes that increase the risk of future reinterventions. Hence, CAD is a significant predictor of decreased time to reintervention.

PAD is associated with a twofold increase in the relative risk of CHF (Meltzer et al. 2012), and patients who progressed from claudication to CLTI were more likely to have CHF (Kim et al. 2020). Having concurrent CHF and PAD portends a worse overall prognosis (Keswani and White 2014). Our study found that CHF is a significant predictor of 12-month AFS. Haemodynamic and physiologic changes in CHF put patients at increased risk of diminished patency following endovascular treatment. Single-centre data in Malaysia and Singapore have shown that the prevalence of symptomatic heart failure in Southeast Asian countries is higher as compared to the rest of the world (4.5–6.7% versus 0.5–2% respectively) (Lam 2015). In a German population, patients with CHF and R6 disease have an increased risk of amputation over long-term follow-up (HR = 1.10) (Freisinger et al. 2017). This risk is just as, if not more, pertinent in Southeast Asian populations. Overall, concomitant CLTI and CHF is associated with poor long-term survival outcomes (Khaira et al. 2017).

CKD is a state of global inflammation which exacerbates vascular calcification and endothelial dysfunction, predisposing a patient to developing PAD (Arinze et al. 2019). The prevalence of CLTI increased with higher stage of CKD (Arinze et al. 2019). PAD and CKD independently predict mortality, but patients with both diseases have a significantly higher risk of death than patients with either alone (Garimella and Hirsch 2014; Liew et al. 2008). In a study of Singaporean diabetics, 17.4% and 12.4% of patients underwent major and minor LEA had CKD respectively, suggesting that CKD may predispose patients to requiring more extensive amputations (Ang et al. 2016). In a German study, patients with CKD had a nearly two-fold higher amputation rate over 4 years (Lüders et al. 2016), and CKD stage 5 was a significant predictor of amputation (Lüders et al. 2016). These associations support our findings that CKD is a significant predictor of 12-month AFS.

CLTI is associated with an appreciable reduction in health-related quality of life and in independence of daily function (Cieri et al. 2011). Co-morbidities such as CHF and CKD are significant predictors of certain treatment outcomes, but R6 disease compounds the negative effects of these co-morbidities on treatment outcomes, including mortality, AFS and wound healing status. Hence, some argue that multiple rounds of endovascular revascularisation for R6 patients would not be the best use of time and resources in view of the poor pre-morbid status of this population and the less-than-ideal treatment outcomes. However, our results show that relatively good results can be achieved with endovascular revascularisation for these patients despite a high rate of reinterventions. Similar AFS and mortality outcomes have been noted in other populations of R6 patients with comparable demographic trends. Hence, there is a role for endovascular intervention instead of a major LEA-first policy, but there may be a subgroup of patients who may benefit from a major LEA-first policy or even a conservative approach.

The limitations of this study include its retrospective design with the associated selection and information biases. It is also a single-centre report subject to abstraction and inherent bias with treatment. Furthermore, we did not obtain information regarding the changes in ambulatory status of the patients post-intervention. If participants were still unable to ambulate post-intervention in spite of complete wound healing, it would mean that revascularisation was a technical success but not a functional success. Additionally, this study did not include quantifiable methods of quality of life and frailty measures, both of which have significant associations with several factors in our study.

## Conclusions

PTA for R6 CLTI patients was associated with relatively high mortality and reintervention rates at one year. Despite multiple rounds of PTA, wound healing rates were acceptable in this challenging group of patients. CAD was an independent predictor of reintervention. More research is required to determine which CLTI patients, especially those with R6 disease, would benefit from an endovascular-first approach versus an immediate major LEA or a conservative policy.

## Data Availability

The datasets generated and/or analysed during the current study are not publicly available due to the Singhealth Centralised Instituitional Review Board requirements but are avaialble from the corresponding author on reasonable request.

## Abbreviations

CLTI: Chronic limb threatening ischaemia
PAD: Peripheral artery disease
LEA: Lower extremity amputation
CVD: Cerebrovascular disease
CAD: Coronary artery disease
CKD: Chronic kidney disease
ESRF: End stage renal failure
DM: Diabetes mellitus
PTA: Percutaneous transluminal angioplasty
VQI: Vascular Quality Initiative
WIfI: Wound Ischemia and foot Infection
TASC II: Trans-Atlantic Inter-Society Consensus Document II
